# Rochester Infants’ Summer Hospital

**Published:** 1889-09

**Authors:** 


ROCHESTER has done herself great credit in establishing an Infants' Summer Hospital at Charlotte, on Lake Ontario, which is due to the munificence of the public-spirited and philanthropic citizens of that enterprising city. Dr. E. M. Moore is President of the Association, and Dr. George W. Goler is the Attending Physician. There are accommodations for twenty children, and any child suffering from intestinal disorder is treated free of charge. Let Buffalo follow this praiseworthy example.

